# Supplementary material for: Leptin, Ghrelin, and Leptin/Ghrelin Ratio in Critically Ill Patients
Source: Nutrients. 2019 Dec 21;12(1):36. doi: 10.3390/nu12010036 (PMC7020071; doi:10.3390/nu12010036)
Supplement: Supplementary file 1 [file nutrients-12-00036-s001.pdf]

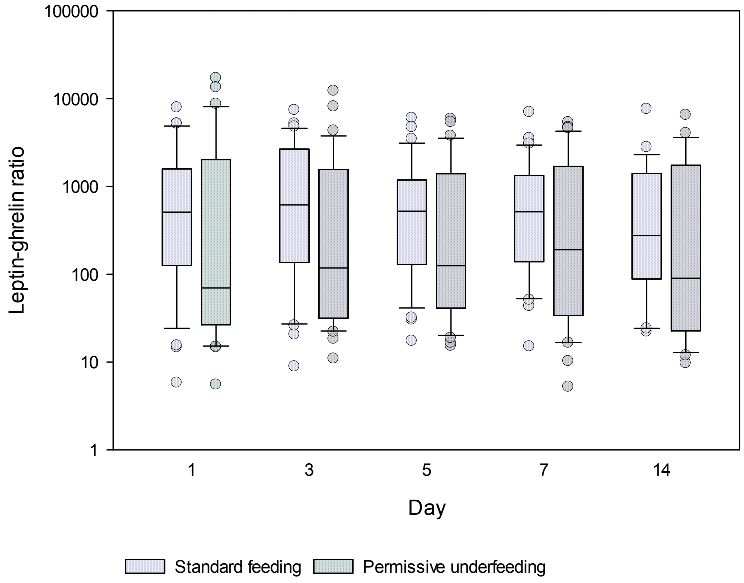

Figure 1. Serial leptin-ghrelin ratio in the permissive underfeeding and standard feeding groups. Data are presented using the box plot with medians and quartiles 1 and 3. The error bars refer to 10th and 90th percentiles.

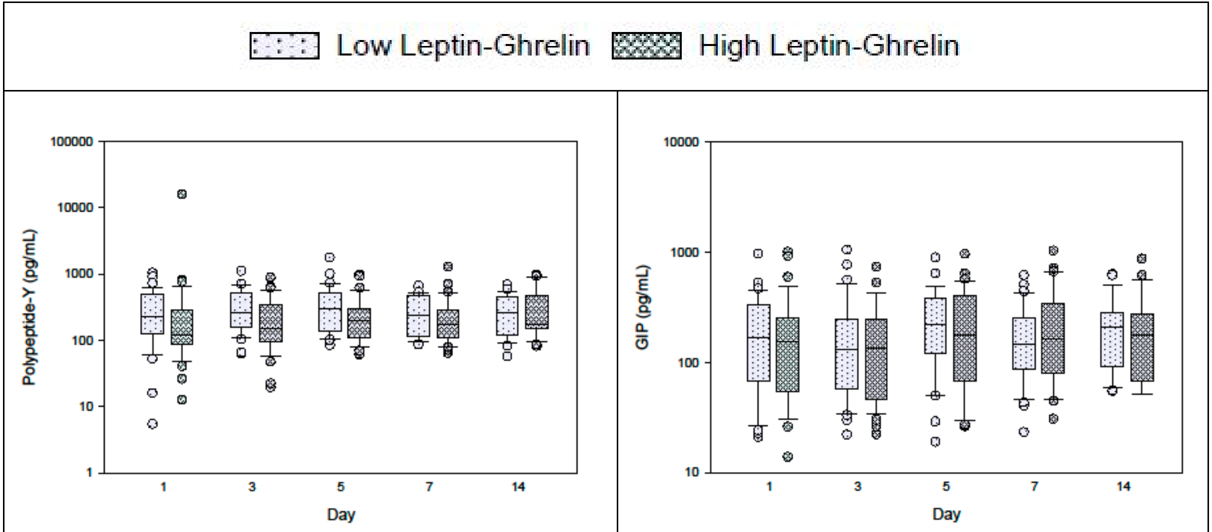

P= 0.78 for between-group difference

P= 0.61 for change over time

P= 0.53 for between-group difference over time

P= 0.87 for between-group difference

P= 0.36 for change over time

P= 0.45 for between-group difference over time

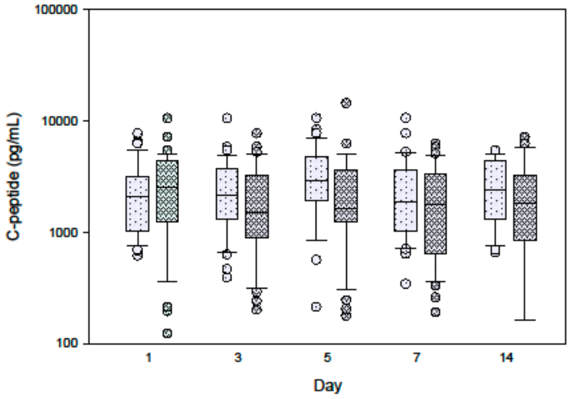

P= 0.39 for between-group difference

P= 0.12 for change over time

P= 0.20 for between-group difference over time

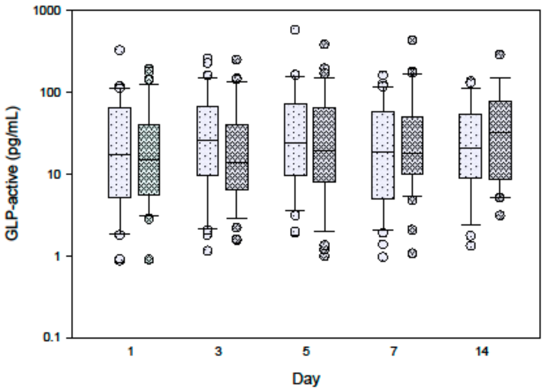

P= 0.99 for between-group difference

P= 0.16 for change over time

P= 0.12 for between-group difference over time

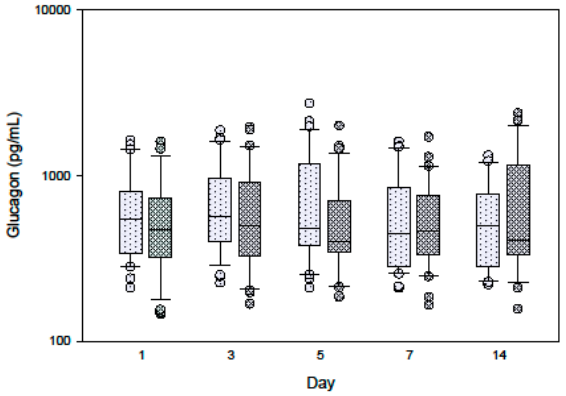

P= 0.57 for between-group difference

P= 0.004 for change over time

P= 0.007 for between-group difference over time

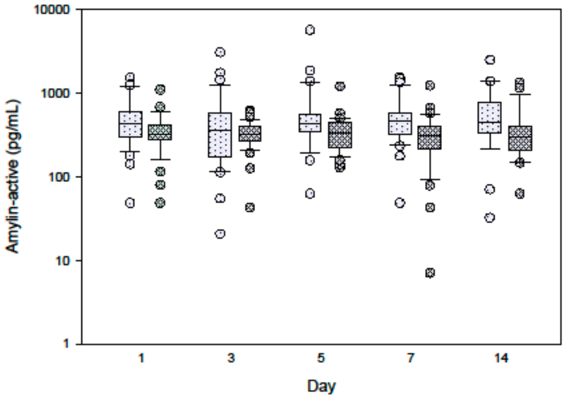

P= 0.008 for between-group difference

P= 0.50 for change over time

P= 0.58 for between-group difference over time

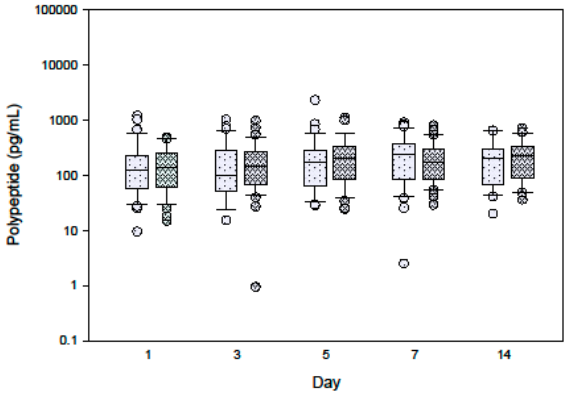

P= 0.91 for between-group difference

P= 0.06 for change over time

P= 0.77 for between-group difference over time

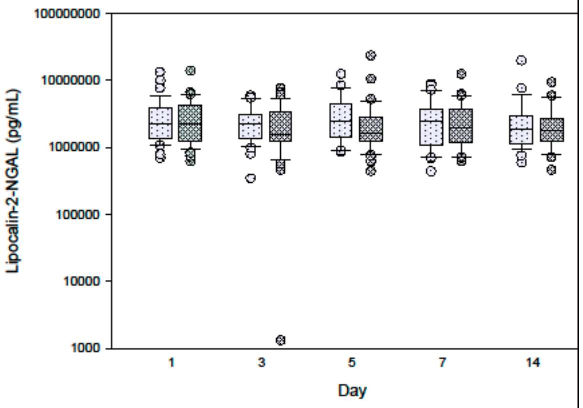

P= 0.57 for between-group difference

P= 0.30 for change over time

P= 0.97 for between-group difference over time

Figure 2. Serial measurements for gut hormones, pancreatic hormones and glycoprotein- lipocalin-2-NGAL in patients with low and high leptin-ghrelin ratio. The differences between groups, with time and between groups with time (group*time) were tested by repeated measures mixed linear models. Box plots are displayed with medians and quartiles 1 and 3. The error bars refer to 10th and 90th percentiles.

Low Leptin-Ghrelin   High Leptin-Ghrelin

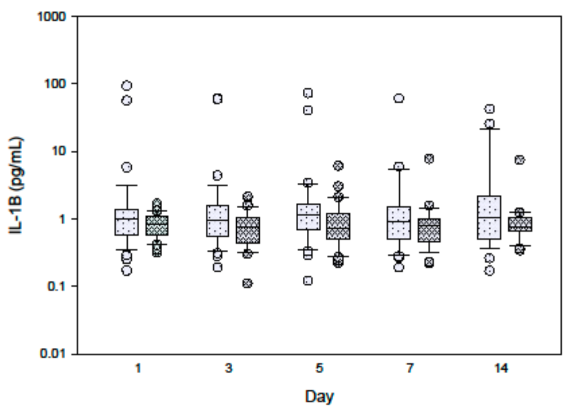

P=0.15 for between-group difference

P= 0.79 for change over time

P= 0.84 for between-group difference over time

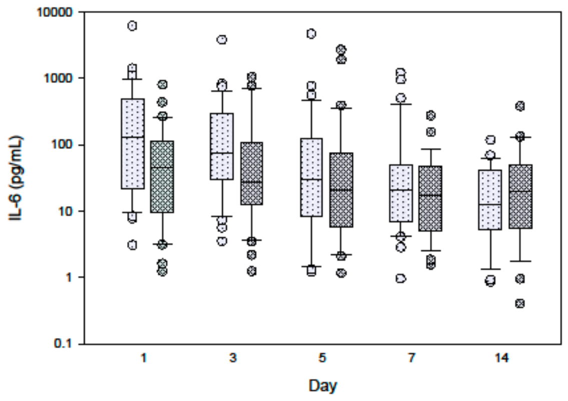

P= 0.11 for between-group difference

P= 0.08 for change over time

P= 0.38 for between-group difference over time

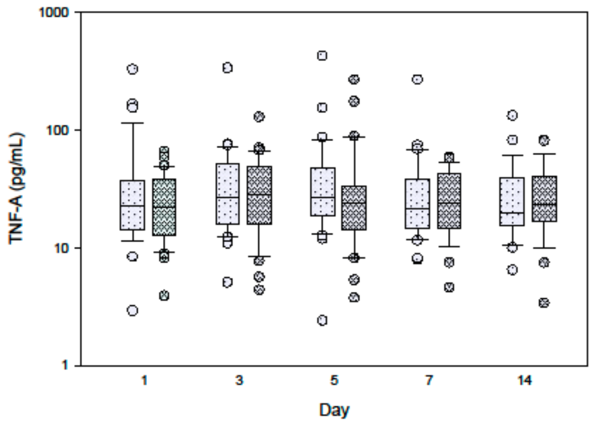

P= 0.74 for between-group difference

P= 0.03 for change over time

P= 0.37 for between-group difference over time

Figure 3. Serial measurements for inflammatory markers in patients with low and high leptin-ghrelin ratio. The differences between groups, with time and between groups with time (group*time) were tested by repeated measures mixed linear models. Box plots are displayed with medians and quartiles 1 and 3. The error bars refer to 10th and 90th percentiles.
